# Person-centred care for migrants: a narrative review of healthcare literature

**DOI:** 10.3389/frhs.2025.1573813

**Published:** 2025-07-16

**Authors:** Cathy Son, Emma Forsgren, Joakim Öhlén, Richard Sawatzky

**Affiliations:** ^1^ School of Nursing, Trinity Western University, Langley, BC, Canada; ^2^ Institute of Health and Care Sciences, Sahlgrenska Academy, University of Gothenburg, Gothenburg, Sweden; ^3^ University of Gothenburg Centre for Person-Centred Care (GPCC), University of Gothenburg, Gothenburg, Sweden; ^4^ Palliative Centre, Sahlgrenska University Hospital, Gothenburg, Sweden; ^5^ Centre for Health Evaluation and Outcome Sciences, Providence Health Care, Vancouver, BC, Canada

**Keywords:** person-centred care, cross-cultural care, interculturalism, migrants, ethnicity, culturally sensitive care

## Abstract

According to the World Migration Report, the number of international migrants has steadily increased in the past 50 years. This has led to an increasing need for healthcare to incorporate a variety of perspectives for migrants. However, healthcare systems still show gaps in accommodating diverse cultural perspectives. Given the increasing attention to person-centred care, there is both an opportunity and a need to explicate how person-centred care (PCC) can help to improve healthcare for migrants. Therefore, we conducted a narrative literature review on cultural dimensions of PCC practice for migrants. A scoping review by Forsgren et al. (2025) identified 1,351 articles from a search of PubMed, Scopus, PsycINFO, CINAHL, and Web of Science databases. From these, nine studies that met the following inclusion criteria were selected: (1) about cultural dimensions of health care for migrants (immigrants and refugees), (2) in any health care settings, (3) written in English, and (4) published within the last 10 years (January 1, 2023-December 31, 2023). The studies included participants from diverse ethnicities, racial backgrounds, and countries of origin. Seven studies were undertaken in primary care, long-term care, or outpatient clinics; one study was on health education; and one additional study focused on the acute care environment. The review led to three main practices: (a) enhancing migrants’ ability to participate in their healthcare, (b) building intercultural partnerships, and (c) promoting cultural education of healthcare providers. These practices underscore the significance of respecting diverse cultural beliefs about shared decision-making and understanding how PCC practice is perceived in different cultural contexts. The results also indicate a need for educational programs that equip healthcare providers with intercultural communication skills and knowledge to provide culturally sensitive PCC. Overall, this study highlights the importance of integrating PCC with interculturalism as a way to foster a more nuanced and responsive understanding of the cultural dimension of care.

## Introduction

Throughout the 20th century, many countries have undergone demographic changes in social structures, economics, and politics. These changes have led to a worldwide increase in migrants; i.e., people living outside their country of birth, including refugees and asylum seekers (1). According to the 2020 World Migration Report, the number of international migrants has steadily increased over the past 50 years, with more than 281 million people living outside their country of origin. This number has more than doubled since 1990, and migrants account for 3.6% of the world's population (2), with 37.6 million refugees living in foreign countries (3). Given the global financial crisis, climate change, and advancements in transportation and communication technologies, a new wave of migration is expected to occur globally (4). This wave of migration is likely to lead to an increased demand for healthcare services among migrant populations. However, there are still significant gaps in healthcare for migrants (5, 6). This includes major challenges faced by migrants that arise due to cultural differences that influence their interactions with healthcare providers, which often hinders their ability to receive appropriate care. The UCL–Lancet Commission on Migration and Health defined culture as “a linked group of customs, practices, and beliefs jointly held by individuals, social networks, and groups. These factors help define who they are, where they stand in relation to those within and beyond the group, and give meaning and order to life” (7). In a policy brief on Cultural Contexts of Health and Well-being, the World Health Organization further explicated the dynamic interrelatedness of culture and health, where culture is understood as “not a static set of beliefs and practices, but rather an ever-emerging array of collective values, ethics, assumptions and ideals” (8).

There are both structural and interpersonal challenges that migrants encounter when receiving healthcare, including the difficulties migrants experience in adapting to a different healthcare system and while also coping with cultural misunderstanding and bias. For instance, Muslim women in North American countries often experience discrimination, insensitivity, and healthcare providers' lack of knowledge about their religious and cultural practices during their healthcare visits (9–12). These negative experiences can likely lead to undesirable consequences, such as avoiding follow-up appointments and facing difficulty managing chronic conditions (13). In addition, research shows that the stress of adapting to the healthcare system can increase the risk of cardiovascular and mental health conditions (14–16). Therefore, addressing both structural and interpersonal aspects of cultural difference is essential for mitigating challenges that migrants may experience (17, 18).

Person-centred Care (PCC) offers one approach to embracing cultural differences by prioritizing the holistic treatment of individuals and empowering them to participate in their own healthcare decisions. PCC foregrounds human dignity and autonomy, stressing the rights of patients as decision-makers in their care process (19). Thus, it challenges the traditional view of patients as mere recipients of healthcare by emphasizing partnership and shared decision-making that is grounded within the patient's narrative, preferences, beliefs, and values (20–22). PCC encourages a comprehensive approach, taking into account the patient's societal and cultural context to identify the most appropriate treatment and care (23, 24). This approach presents an opportunity to embrace cultural differences and promote optimal healthcare for migrants.

Although PCC can offer various benefits, there may also be unintended consequences. Concerns have been raised that PCC is grounded in individualistic values that may not be suitable for people who have inherited collectivist cultural values (25, 26). Much of the PCC literature reflects a Westernized and individualistic cultural context. This raises critical questions about the cultural adaptability of PCC and how PCC can meet the healthcare needs of migrants who have diverse cultural backgrounds. In cultures where the decision is made by the community or by the family, the PCC's emphasis on individual autonomy may not align with their cultural values (25, 27). The potential for PCC as an approach to better meet the healthcare needs of migrants, therefore, warrants critical examination.

To further explore this opportunity, we conducted a review of studies that offer insights into the cultural dimensions of PCC for migrants. Our review question was: How have PCC involving cultural dimensions of care for migrants been practiced?

## Method

We employed a narrative review approach to critically analyze and interpret cultural aspects of PCC. Narrative reviews are often used to provide summaries and analyses of literature on specific topics to introduce theories or diverse viewpoints, enabling the exploration of lesser-studied areas (28). This is accomplished by synthesizing various literary sources to provide a multifaceted understanding of a topic and offer critiques of prior studies, which is crucial in establishing directions for future research (29, 30). Considering the lack of empirical studies available, a narrative review allows for a flexible approach to include diverse perspectives that may not meet stringent inclusion criteria of structured review types (30). To enhance the rigour of this study, the Scale for the Assessment of Narrative Review Articles (SANRA) was used to ensure that all aspects of the review process were thoroughly covered (31).

Our review builds on an overarching scoping review of international literature on PCC conducted by Forsgren et al. (32) Forsgren et al. included different terms referring to the concept of PCC, such as “people-centred”, “family-centred”, and “patient-centered”, variations on term endings (e.g., centric, centeredness) and accompanying terms (e.g., care, practice, approach) to capture the broad range of relevant studies. The core element of the PCC is that patients are recognized as individuals with unique insights into their health conditions and integral members of the healthcare team alongside professionals and other significant individuals in the patient's life. The scoping review involved a comprehensive search of index terms and free text words related to PCC in several databases, which resulted in 94,236 citations. Based on the above definition and corresponding inclusion criteria, Forsgren et al. identified 1,351 relevant articles by employing a methodology that combined manual and text-mining screening.

The 1,351 articles were screened by CS in ongoing consultation with all co-authors to identify studies that were: (1) about cultural dimensions of healthcare for migrants (immigrants and refugees), (2) in any healthcare setting, (3) written in English, and (4) published within the last 10 years (January 1, 2013–December 31, 2023). The search was limited to the past 10 years to ensure relevancy to the current practices and migration contexts, given the rapid changes in healthcare delivery and migration landscape in recent years. Exclusion criteria were: (1) reports about theories, theoretical models, or frameworks, (2) review studies that did not include original data, (3) studies on cross-cultural topics that did not involve migrants as subjects of the study, and (4) studies that focused on social determinants other than migrant status. After an initial screening of titles and abstracts, CS conducted a full-text review of all potentially relevant citations. The screening results were discussed and verified with all co-authors via regular meetings through which consensus on the final set of included studies was achieved. As a result, nine studies were included in the analysis. The flow diagram summarizes the reason for selecting these nine studies (Supplementary Figure S1, Adapted PRISMA flow diagram).

The EPPI-Reviewer software was used for coding and data extraction of the included studies, which was led by CS in ongoing consultation with all co-authors. The nine relevant studies were organized into a tabular format, including publication year, first author, country, title, study purpose, study area, participants' ethnicities, and research methods (Table 1). Each study was read multiple times to extract and organize data relevant to the review questions. Following an interpretive approach, the synthesis involved constructing key themes informed by critical reflections by all co-authors and theoretical underpinnings of PCC; subsequently, the themes were interpreted as overall PCC practices that involve cultural dimensions of care.

**Table 1:** Characteristics of articles reviewed for cross-cultural aspects of PCC.

Author Year Title	Purpose	Care setting Country	Target population	Research methods
Bentwich et al. (2018) How figurative language may be related to formal caregivers’ person-centred approach toward their patients with dementia	To explore how figurative language may be related to formal caregivers’ person-centred approach toward their patients with dementia	Long-term Care, Israel	Immigrant caregivers from the Soviet Union, Jews born in Israel, and Arabs (*n* = 20)	Qualitative study Interviews
Durante (2016) Family-centered Care as a predictor of early intervention outcomes for ethnically diverse families	To examine the experiences of racially and ethnically diverse families in early intervention with regard to family-centered service delivery	Public Health, USA	Caucasian, African American, and Hispanic families with children who are less than 31 months of age (*n* = 3,338)	Quantitative study Secondary analysis
Guerrero et al. (2010) Racial and ethnic disparities in pediatric experiences of family-centered Care	To examine racial and ethnic disparities in the receipt of family-centered Care among a general population of US children	Public Health, USA	White, black, Latino, and other race children (*n* = 4,278)	Quantitative study Survey
Ingram et al. (2015) Using participatory methods to enhance patient-centred mental health care in a federally qualified community health center serving a Mexican American farmworker community	To assess and address gaps in perceptions of mental healthcare between providers and migrant workers living in a US–Mexico Border community	Primary Care, USA	Mexican American farmworkers and federally qualified community health center staff (*n* = 80)	Qualitative study Focus groups and interviews
Montes & Halterman (2011) White-Black disparities in family-centered Care among children with autism in the United States: Evidence from the NS-CSHCN 2005–2006	To compare the reported receipt of family-centred Care between parents of white and black children with autism spectrum disorder (ASD) in the United States	Public Health, USA	Parents and guardians of white and black children with ASD (*n* = 35,386)	Quantitative study Secondary analysis
Tucker et al. (2011) Patient-centered, culturally sensitive healthcare	To explain and improve healthcare for ethnically diverse patients seen in community-based primary care clinics	Primary Care, USA	African American and non-Hispanic White Americans (*n* = 229)	Quantitative study Survey
Wall et al. (2013) ‘Patients’ perceived cultural sensitivity of health care office staff and its association with ‘patients’ health care satisfaction and treatment adherence	To examine that patient-perceived cultural sensitivity of front desk office staff has a significant positive association with patient treatment adherence	Primary Care, USA	White American, African American, Hispanic, American Indian/Native American, Asian/Asian, American/Pacific Islander, other race ethnicity (*n* = 1,191)	Quantitative study Survey
Watt et al. (2013) Family-centred Care: A qualitative study of Chinese and South Asian immigrant parents’ experiences of Care in pediatric oncology	To describe Chinese and South Asian immigrant ‘parents’ experiences of Family-centred care in pediatric oncology settings in Canada	Acute Care, Canada	First-generation Chinese and South Asian parents of children with cancer who were at least 6 months post-cancer diagnosis (*n* = 50)	Qualitative study Interviews
Wilkerson et al. (2010) Assessing patient-centered care: One approach to health disparities education	To compare the reliability, validity, and feasibility of an embedded patient-centred care scale with the use of a single culturally challenging case in measuring students’ use of person-centred care behaviours as part of a comprehensive objective structured clinical examination (OSCE)	Health education, USA	Senior medical students at two California medical schools (*n* = 322)	Quantitative study Observational study

## Results

Of the nine original studies, seven were conducted in the United States, one in Canada, and one in Israel. Regarding healthcare settings, seven studies were conducted in primary care, long-term care, or outpatient clinics, and the remaining studies were conducted in medical school acute care settings. Migrants (i.e., those born outside of the host country) in the included studies exhibited a diverse range of ethnicities, racial backgrounds, and countries of origin, including Caucasian, African American, Hispanic, American Indian, Asian/Asian American/Pacific Islander, Chinese, South Asian, individuals from the former Soviet Union, Jews born in Israel, and Arabs. The studies covered a broad range of participants, including patients (covering both children and adults), healthcare providers and staff, parents or family members, and students (see Table 1).

Culture in the studies reviewed has been conceptualized in distinctive ways. Culture is understood both as the characteristics of a specific group with shared beliefs, values, customs, and behaviours, and also as a dynamic construct that is shaped by social interactions and that changes over time (33, 34). The included studies on PCC reflect an evolving understanding of culture that highlights the importance of being responsive to the fluid and diverse cultural identities that migrants bring to healthcare experiences. This aligns with the fundamental concept of PCC, which embraces responsiveness to individuals' unique experiences and the diverse cultures that migrants bring to healthcare. Building on these understandings, the synthesis identified the following three main practices about PCC for migrants: (a) enhancing migrants' ability to participate in their healthcare, (b) building intercultural partnerships, and (c) promoting cultural education of healthcare providers.

### Enhancing migrants' involvement in their healthcare

Migrants often face language barriers and communication challenges. These barriers can hinder their ability to clearly communicate their experiences as well as healthcare preferences, which can reduce their ability to participate in their healthcare (35, 36). Overcoming language barriers and meeting the linguistic needs of migrants is vital to ensure that they receive adequate care (36). Receiving care in migrants' native language can enhance a shared understanding of health and foster a sense of respect from healthcare providers (37). The reviewed studies recommend a variety of strategies to improve communication with migrants facing language barriers including: (a) providing clear action-oriented steps, assessing migrants' comprehension, (b) using interactive communication loops, (c) reducing the use of medical terminology, and (d) employing medical interpreters are recommended strategies (36, 37). PCC studies also propose that educational materials for migrants be supplemented with culturally and linguistically appropriate content to ensure that patients receive more relevant and accurate information (37). These approaches not only mitigate language barriers but also empower migrants to take an active role in their healthcare. By adopting these approaches, PCC can encourage healthcare providers to uphold migrants’ rights and dignity by promoting their participation in decision-making (34, 36, 38).

### Building intercultural partnerships

PCC highlights the importance of building intercultural partnerships, where individuals from diverse cultural backgrounds work together through mutual understanding and collaboration. To promote this partnership, PCC studies note the importance of developing cultural sensitivity. Cultural sensitivity is more than simply recognizing the unique cultural experiences of migrants; it involves respecting and responding to their unique cultural needs (34). Cultural sensitivity can be achieved by healthcare providers paying close attention to the expectations of migrants and being responsive to the unique attitudes, emotions, and situations of migrants (33, 36, 38, 39). This includes allocating sufficient time to provide medical information and building trust through emotional empathy and attentiveness (34–36, 38, 39). Culturally sensitive care increases migrants' trust and comfort, leading to positive health outcomes and satisfaction (37). This approach can also improve the ability of migrants to manage their interpersonal interactions and take ownership of their healthcare.

PCC studies also draw attention to the need for understanding that migrants are not a homogeneous group with uniform needs. Each migrant faces healthcare issues and circumstances contextually (35, 39). If there are discrepancies between how migrants perceive illness and the explanation provided by healthcare providers, migrants may lose motivation to follow the suggested treatment regimen (33, 35). Thus, healthcare providers should continually assess whether their practices are aligned with migrants' unique cultural contexts (35, 36, 40). This practice requires healthcare providers to shift away from Western-centric models of care towards embracing culturally responsive approaches (40).

PCC studies also point out that healthcare providers must avoid imposing decision-making roles on migrants that may not be aligned with their cultural beliefs and norms. For example, some cultures may take a family decision-making process, whereas others may take a community-based decision-making process (40). As well, in some families, the patient will make their own decision, whereas others will not, and this is often determined by culture. Therefore, clear expectations should be set early on through transparent communication, allowing migrants to be involved in the decision-making process in a way that is consistent with their cultural norms (38, 40). The PCC studies show that the more opportunities migrants have to exercise control over their care, the more inclusive and culturally sensitive care can be achieved (40).

### Promoting cultural education of healthcare providers

PCC studies underscore the significance of cultural education for healthcare providers. By learning about different cultures, healthcare providers can broaden their understanding of diverse cultural needs (33, 34, 37). PCC studies emphasize that cultural education should go beyond acquiring knowledge about different cultures. Rather, cultural education should foster self-assessment and reflection, awareness of cultural diversity, and adaptability to different cultural contexts (33, 35, 38, 39). Specifically, cultural education should enhance the providers' ability to engage in culturally sensitive interactions with migrants. This includes developing healthcare professionals' skills in communication and counselling, which are essential for eliciting migrants' personal narratives (33, 36, 38). Furthermore, cultural education should be a resource for healthcare providers to become aware of cross-cultural barriers, such as implicit bias, health disparities, and inequities (36–38). All healthcare workers who provide services to migrants and their communities, not only healthcare professionals, need cultural education. Thus, this education should be tailored to the specific roles of healthcare workers to maximize its effectiveness (34, 35).

## Discussion

This review was conducted to offer insights into the application of cross-cultural PCC for migrants. We identified several important practices accentuating the need to: (a) enhance migrants' ability to participate in their healthcare, (b) build intercultural partnerships, and (c) promote cultural education of healthcare providers. The results further draw attention to the importance of distinguishing intercultural care from cross-cultural approaches.

Cross-cultural approaches and PCC share commonalities in acknowledging cultural diversity and aim to meet the needs of all people (41). Both approaches underscore the values, preferences, languages, and traditions of migrants as important considerations in their care. However, there are also notable differences. Cross-cultural approaches tend to view migrants as a collective group and support the notion of culture as a set of shared values (42). In contrast, PCC tends to view each migrant as a person with a unique experience and focuses more on individual narratives (27). As such, PCC seeks to promote an individualist approach that integrates migrants' personal stories into healthcare decision-making, thereby ensuring that treatments are tailored to individuals. In this regard, bringing together PCC and interculturalism may provide an alternative approach to developing a more nuanced understanding of migrant care.

Interculturalism is an approach that extends beyond merely acknowledging different cultural groups, as it places greater emphasis on interaction and understanding between cultures. Unlike deterministic perspectives that view cultures as fixed identities defined by geographical boundaries, interculturalism highlights that culture is dynamic and continuously evolving (41, 43, 44). It also acknowledges that individuals may have multiple cultural identities. Interculturalism encourages harmonious coexistence among individuals from diverse cultural backgrounds by shifting focus from differences to commonalities (43). Both interculturalism and PCC share the understanding that culture can be influenced by various intersectional factors. Thus, no two migrants’ experiences can be the same (26, 45, 46). This perspective supports that healthcare requires a flexible and tailored approach to care that respects each migrants' unique cultural and personal circumstances.

Established PCC frameworks, such as McCormack and McCance's Person-Centred Practice Framework and the University of Gothenburg Centre for Person-Centred Care (GPCC) framework, align with the core value of interculturalism. They draw attention to the importance of understanding individual narratives and context, respecting beliefs and values, and fostering partnerships between patients and healthcare providers (47, 48). However, these frameworks still lack explicit attention to cultural diversity. In this regard, there is an opportunity to integrate interculturalism in PCC frameworks with the goal of developing a more nuanced understanding of culturally sensitive healthcare for migrants. When integrated with an intercultural perspective, PCC requires a foundation of self-reflection on cultural biases and stereotypes, an appreciation of diversity, and finding common ground as a basis for meaningful interaction and relationships. In this approach, no cultural perspective is considered superior, and cultural humility, or respecting others' cultural values, becomes the foundation (49). Additionally, this integration requires individuals to take responsibility for continually recognizing their own cultural biases while striving to form equitable partnerships with migrants (50). This demands a flexible mindset, concern for both self and others, and a belief in the inherent equal value of all human beings (51).

Integrating interculturalism into healthcare requires effective communication that acknowledges and navigates culturally shaped beliefs and practices. This can be facilitated by implementing education about intercultural communication to enhance cultural sensitivity and improve how healthcare providers interact with migrants. This requires a well-balanced approach to education around three key components: cognitive, affective, and behavioural (52–55). The cognitive component involves the ability to understand and correctly interpret both verbal and nonverbal messages. The affective component involves the ability to empathize with and respect the emotions and lived experiences of individuals from different cultural backgrounds (53, 54). The behavioural component refers to how effectively and appropriately individuals apply the cognitive and affective components in real-life interactions (56). For example, education on intercultural communication could include interactive training sessions for providers to practice and engage in discussions about how to improve cognitive awareness (e.g., interpreting body language), affective response (e.g., demonstrating empathy) and behaviour strategies (e.g., adapting communication style) (53–55).

The findings of this review also provide insights into potential system-level changes to support PCC for migrants. Clearly, it is important for the voices of migrants to be represented when making changes that affect their healthcare services. Interculturalism emphasizes that cultural influences are not unidirectional, but rather multidirectional (57). This means ensuring that the perspectives and practices of migrants are valued in healthcare services and systems. It is particularly important to recognize migrants as agents, not only by listening to their needs, but by supporting migrant-led improvements to the care they receive. To enhance migrants' involvement and build intercultural partnerships, healthcare organizations and policymakers may consider implementing intercultural mediators who can help to reduce cultural misunderstandings while ensuring that migrants receive healthcare that aligns with their culturally shaped views of health and healthcare (58, 59). Intercultural mediators are professionals with training in understanding cultural differences and representing the perspectives of all cultures in healthcare delivery (58–60). In this capacity, their role is not limited only to finding common ground for communication, but also to promoting balanced power dynamics so that care decisions affecting migrants do not solely reflect the healthcare professional's advice.

## Limitation of the study

This review was based on nine studies, seven of which were conducted in the United States one in Canada, and one in Israel, and should, therefore, not be regarded as representative of healthcare for migrants globally. Additionally, the review was based on studies conducted within several distinct healthcare settings. Thus, PCC practices across different care contexts are not represented in the synthesis. Given the small number of studies identified and considering the limited representation of different healthcare systems (i.e., predominantly in the United States of America), there is a significant opportunity for further research and theoretical development on PCC for migrants by integrating interculturalism into existing PCC frameworks and practices.

The selection of studies was based on a database of research explicitly inquiring about PCC as a concept (including various terms for the concept). Limiting the scope to PCC studies may have excluded valuable insights from other studies that investigated cultural aspects of migrant healthcare. Future research could expand on this work by examining how PCC relates to other theoretical frameworks and approaches to migrant care.

## Conclusion

As the global migrant population continues to grow, the demand for culturally inclusive care has increased in healthcare. This narrative review study explores the cultural dimension of PCC in the context of migrants' care and identifies three significant practices. First, PCC prioritizes empowering migrants to be actively involved in their healthcare decision-making as well as striving to reduce language barriers. PCC also points to the importance of building intercultural partnerships through cultural sensitivity and respecting migrants' culturally shaped beliefs and experiences. Lastly, PCC requires strengthening cultural education by focusing on meaningful interaction. The findings point to an opportunity to integrate PCC and interculturalism as an approach to promote inclusivity and cultural sensitivity in healthcare, with the goal to foster cultural humility among healthcare providers and ultimately improve healthcare outcomes for all people, including migrants.
