# Supplementary material for: Person-centred care for migrants: a narrative review of healthcare literature
Source: Front Health Serv. 2025 Jul 16;5:1573813. doi: 10.3389/frhs.2025.1573813 (PMC12307281; doi:10.3389/frhs.2025.1573813)
Supplement: Supplementary file 1 [file Datasheet1.pdf]

Supplementary Material

Supplementary Figure 1. Adapted PRISMA flow diagram

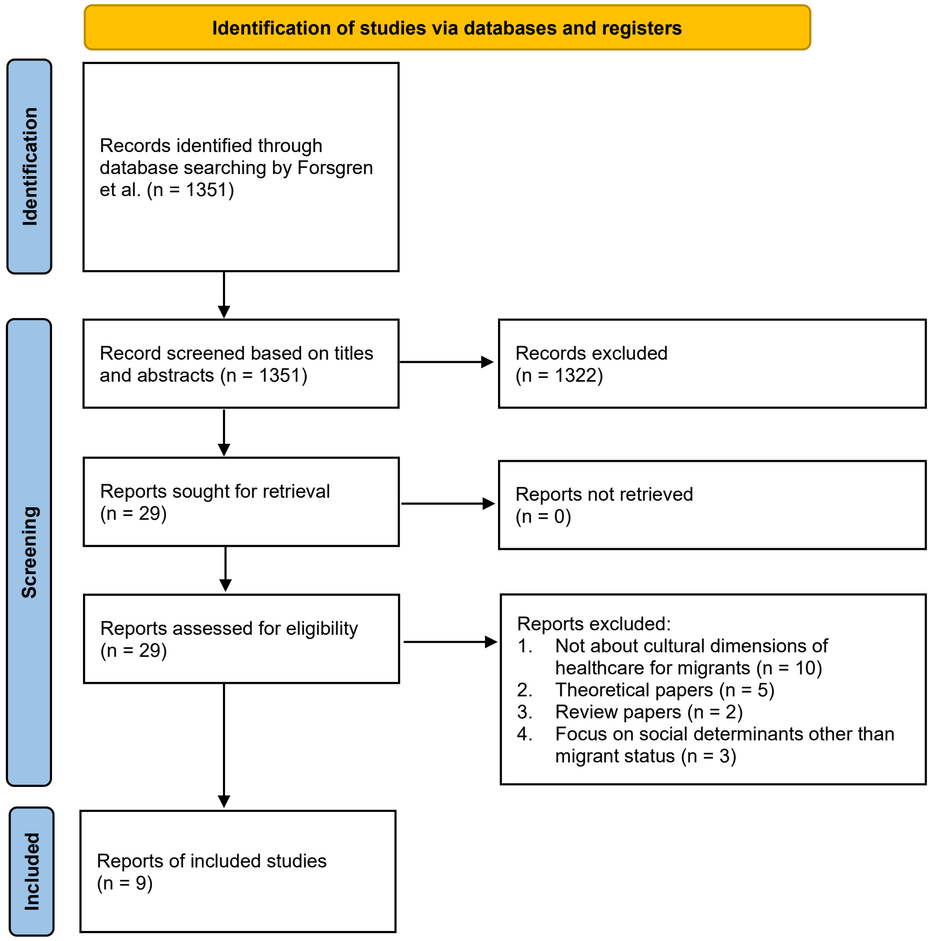

Adapted from: Page M, Moher D, Bossuyt P, Boutron I, Hoffmann T, Mulrow C, et al. PRISMA 2020 explanation and elaboration: updated guidance and exemplars for reporting systematic reviews. BMJ [Internet]. 2021;372. Available from: https://www.bmj.com/content/372/bmj.n160.short
